# Metabolomic Prediction of Human Prostate Cancer Aggressiveness: Magnetic Resonance Spectroscopy of Histologically Benign Tissue

**DOI:** 10.1038/s41598-018-23177-w

**Published:** 2018-03-26

**Authors:** Lindsey A. Vandergrift, Emily A. Decelle, Johannes Kurth, Shulin Wu, Taylor L. Fuss, Elita M. DeFeo, Elkan F. Halpern, Matthias Taupitz, W. Scott McDougal, Aria F. Olumi, Chin-Lee Wu, Leo L. Cheng

**Affiliations:** 1 Department of Pathology, Massachusetts General Hospital, Harvard Medical School, Boston, Massachusetts 02114 USA; 2 Department of Radiology, Massachusetts General Hospital, Harvard Medical School, Boston, Massachusetts 02114 USA; 3 Department of Urology, Massachusetts General Hospital, Harvard Medical School, Boston, Massachusetts 02114 USA; 4 Department of Radiology, Charité Medical University of Berlin, Charitéplatz 1, 10117 Berlin, Germany

## Abstract

Prostate cancer alters cellular metabolism through events potentially preceding cancer morphological formation. Magnetic resonance spectroscopy (MRS)-based metabolomics of histologically-benign tissues from cancerous prostates can predict disease aggressiveness, offering clinically-translatable prognostic information. This retrospective study of 185 patients (2002–2009) included prostate tissues from prostatectomies (n = 365), benign prostatic hyperplasia (BPH) (n = 15), and biopsy cores from cancer-negative patients (n = 14). Tissues were measured with high resolution magic angle spinning (HRMAS) MRS, followed by quantitative histology using the Prognostic Grade Group (PGG) system. Metabolic profiles, measured solely from 338 of 365 histologically-benign tissues from cancerous prostates and divided into training-testing cohorts, could identify tumor grade and stage, and predict recurrence. Specifically, metabolic profiles: (1) show elevated *myo*-inositol, an endogenous tumor suppressor and potential mechanistic therapy target, in patients with highly-aggressive cancer, (2) identify a patient sub-group with *less* aggressive prostate cancer to avoid overtreatment if analysed at biopsy; and (3) subdivide the clinicopathologically indivisible PGG2 group into two distinct Kaplan-Meier recurrence groups, thereby identifying patients more at-risk for recurrence. Such findings, achievable by biopsy or prostatectomy tissue measurement, could inform treatment strategies. Metabolomics information can help transform a morphology-based diagnostic system by invoking cancer biology to improve evaluation of histologically-benign tissues in cancer environments.

## Introduction

The utility of the serum prostate specific antigen (PSA) screening test is now well established, both by its capacity to reveal early-stage disease and its discovery of almost all newly-detected prostate cancers^1–3^. However, despite being *prostate*-specific, PSA testing is not *cancer*-specific. Current medical imaging detects 44–87%^4^ of clinically significant cancers but remains challenged by small lesions. For this reason, prostate cancer (PCa) diagnoses are still only positively confirmed by prostate transrectal biopsies, performed after an elevated PSA result. After a positive biopsy, an initial cancer grade is assigned according to histological analysis of the biopsy cores; this grade will help determine whether the prostate should be surgically removed by prostatectomy or the patient should enter active surveillance. If a prostatectomy is performed, histological examination of the entire prostate will determine the pathological stage of the disease. With this information, follow-up therapies can be recommended, if warranted.

Statistics on prostate cancer’s natural history suggest that >70% of patients diagnosed after PSA screening are likely to experience an indolent disease course that little impacts their well-being. About 17% of these newly PSA-diagnosed patients, however, will confront an aggressive prostate cancer that significantly impairs function and truncates life expectancy^5^. Moreover, the histological presentations of many PCa cases are indistinguishable. In the search for more cancer-specific markers, prostate-specific membrane antigen (PSMA) levels have been studied. However, these studies report mixed results, both for PSMA levels’ utility in correlating with tumor grade and pathological stage^6^ and for their potential benefit in detecting metastases^7^, biochemical recurrence^8^, and monitoring therapy^9^. Thus the clinical challenge still facing the PSA era is to differentiate between prostate cancer patients with indolent tumors versus those who require definitive therapy.

Genomics, proteomics, and metabolomics have responded to prostate cancer clinical challenges by investigating new tumor biomarkers^10,11^. Metabolomics can be seen as the integrated read-out of upstream biochemical activities involving the genome and proteome in disease processes. Metabolomics examines the entire, measurable metabolome to discover global, cancer-specific profiles that have the potential to signal disease dynamics from domains not examined by histology. One of these areas is the increasingly recognized cancer research focus of tumor-stroma interactions and tumor microenvironments, as presented in the context of cancer genomics, proteomics, and transcriptomics^12–17^.

Positing tumor metabolomic microenvironments to be sensitive to prostate cancer characteristics, we investigated the clinical potential of tissue magnetic resonance spectroscopy (MRS) analysis of human histologically-benign (Hb; no histologically identifiable prostate cancer cells or glands) samples that were obtained from cancerous prostates. To discover metabolomic profiles of prostate cancer disease, we used high-resolution magic angle spinning (HRMAS) MRS, which we developed for intact tissue metabolic analysis^18,19^, and the 2016 five-step, International Society of Urological Pathology prostate cancer Prognostic Grade Group (PGG) system^20^, based on clinical data obtained across previous decades and representing the most up-to-date prostate cancer pathology scale evolved from the original Gleason scores.

## Materials and Methods

### Patients

**Figure 1:**
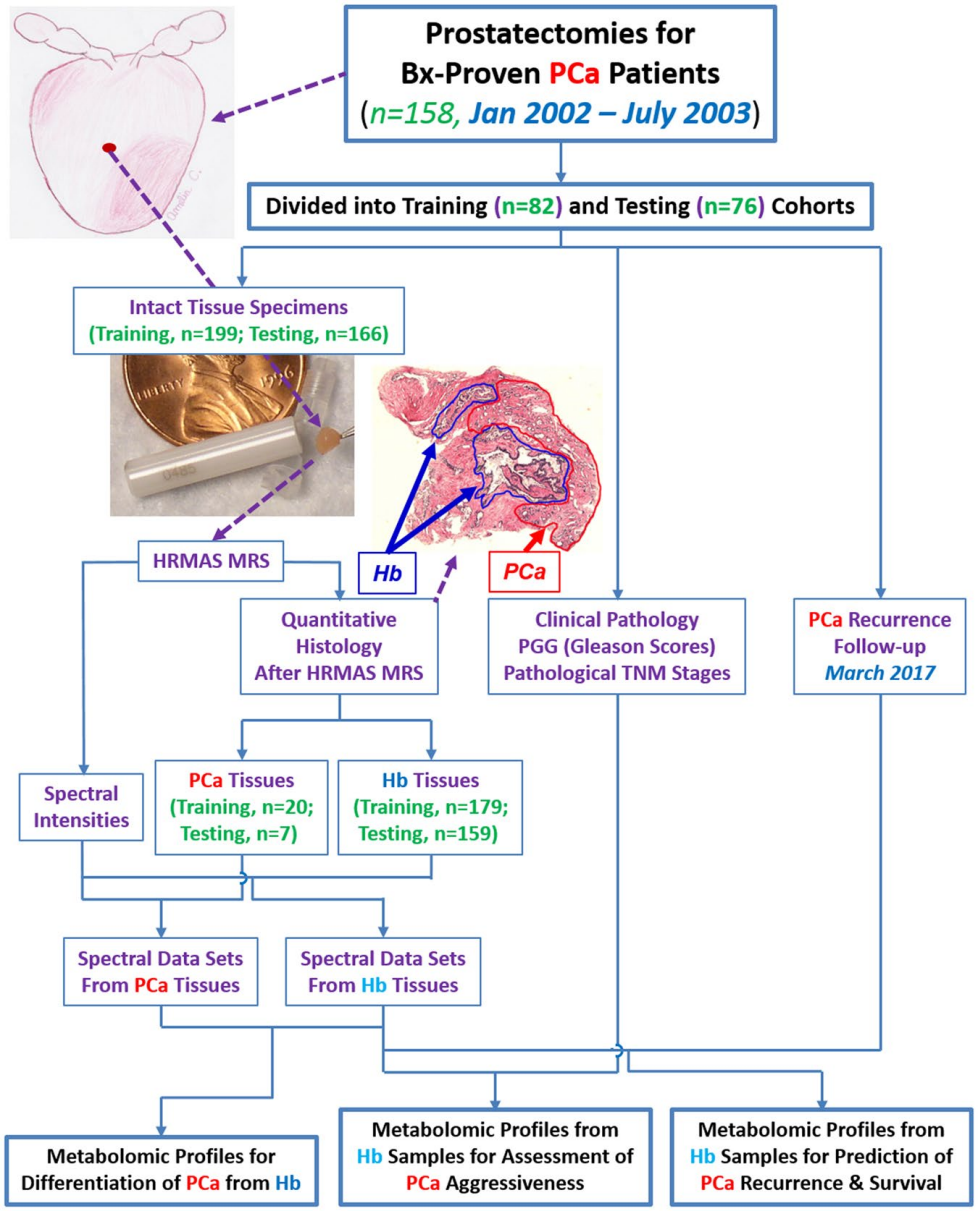
Experimental Design. A representative histology cross-section to demonstrate preservation of histology features after HRMAS MRS. Red and blue lines illustrate cancer and histologically-benign glands, respectively; remaining areas include glands and stroma. Prostate illustration created by Amelia C., 2013.

This retrospective study was approved by the Partners Human Research Committee IRB (2005P000774) and carried out in accordance with the specified guidelines and regulations. Informed, written patient consent was obtained prior to obtaining tissue samples, and patients were informed that the extra tissue banked from their already excised biopsies or prostates would confer no added risks or benefits to them. De-identified surgical samples were obtained from the tumor bank of the MGH Urological Pathology Research Laboratory and, according to standardized protocol, were snap-frozen in capsulated vials in liquid nitrogen <45 minutes after radical prostatectomy, transurethral resection of the prostate, or biopsy (Bx) and stored at −80 °C until analysis. When available, multiple samples from the same prostate were gathered, and a total of 394 tissue samples from 185 patients were analyzed. Specifically, 365 prostatectomy samples, acquired between January 2002 and July 2003 from 158 biopsy-proven prostate cancer patients, were analyzed; the overall experimental design for evaluating samples from prostatectomy is shown in Fig. 1. We randomly divided prostatectomy cases into training (first 199 samples from 82 patients)^11^ and testing (remaining 166 samples from 76 patients) cohorts. Additionally, 15 benign prostatic hyperplasia (BPH) samples from 13 patients who underwent TURP without known presence of prostate cancer, and 14 prostate Bx cores, were collected between April 2006 and February 2009 from 14 patients with elevated PSA readings, but no evidence of prostate cancer to the present time (mean follow-up, 9.2 yrs; range, 8.1–10.9 yrs). The BPH samples were used to investigate potential metabolic contributions from BPH. HRMAS MRS results in Bx specimens from patients who remain cancer-negative after over eight years of follow-up provided ‘conditional controls’ for comparison with HRMAS MRS results in Hb samples from prostate cancer patients.

Patient clinical and pathological information obtained from the UPRL database included: Age, 58.5 ± 6.5 (Max:75; Min:41) yrs; pre-surgical serum PSA, 5.6 ± 3.1 (20.0; 0.3) ng/ml; pre-surgical PSA density, 0.13 ± 0.08 (0.56; 0.01) ng/ml/g; pre-surgical serum percent-free PSA, 15.0 ± 8.4 (45; 4)%; prostate weight, 47.0 ± 1.0 (140.8; 20.8) g; Prostate Grade Group (PGG)^20^: 1 (86), 2 (52), 3 (13), 4&5 (7); American Joint Committee on Ccancer pathological tumor stage of pT, pT = IIab (56), pT = IIc (76) and pT = III (26); and prostate cancer perineural invasion, positive (97) and negative (61). PGG evaluation of tissues containing prostate cancer glands after MRS measurement agreed with patients’ pathological records. For BPH, clinical data are: Age, 65.4 ± 2.7 (Max: 80; Min: 55) yrs; PSA, 4.2 ± 1.1 (11.2; 0.2); and estimated prostate weight, 125.1 ± 15.1 (204; 70) g. For Bx patients with elevated PSA, clinical data are: age, 63.1 ± 2.0 (Max: 74; Min 47) yrs; PSA 6.2 ± 2.6 (11.9; 3.3); and estimated prostate weight, 62.6 ± 8.9 (110, 30) g.

### Intact tissue MRS

Intact tissue MRS was conducted with our previously developed methodology, high-resolution magic angle spinning (HRMAS) on a Bruker (Billerica, MA) AVANCE 600 MHz spectrometer. MRS was performed blinded to the clinical status of samples. Tissues were weighed (~10 mg) and placed into a 4 mm rotor with 10 µl plastic inserts, with 1.0 µl D_2_O (Sigma Aldrich, St. Louis, MO) for field locking. Spectra were recorded at 4 °C, with spectrometer frequency centered on the water resonance, a repetition time of 5 s, and a rotor-synchronized DANTE protocol conducted with spinning at both 600 and 700 (±1.0) Hz^21^.

HRMAS MRS data were analysed with AcornNMR-Nuts (Livermore, CA) and presented in the form of peak intensities for spectral regions, with the unit parts per million (ppm) of magnetic field strength. Spectral intensities were integrals of curve-fittings with Lorentzian-Gaussian line-shapes (Fig. S1). Intensities in 36 spectral regions resulted from spectral curve-fitting for peaks in the 0.5–4.5 ppm spectral range, excluding regions of potential biopsy lubricant gel contamination. Relative spectral intensities for each of the 36 spectral regions represent fitted spectral peak values, normalized by total spectral intensities from 0.5–4.5 ppm. Spectral regions and possible major contributing metabolites for each region, listed in Table S1, were assigned according to the literature^22–24^.

### Quantitative Histopathology

After HRMAS MRS, tissues were fixed in 10% formalin, embedded in paraffin, cut into 5 μm sections at 100 μm intervals throughout the entire sample, and haematoxylin and eosin (H&E) stained. In general, each studied tissue sample (~10 mg) produced from 10 to 16 histological cross-sections. Archived histology slides for all cases were evaluated using the new PGG system^20^. Two genitourinary pathologists, with seventeen and eight years, respectively, of prostate cancer clinical experience and no knowledge of spectroscopic results, visually estimated to the nearest 5% any area representing cancer (including lumens), benign epithelia (including lumens), and stroma in each cross-section for vol% calculations. For the 338 of 365 prostatectomy samples that were determined to be Hb tissue, apart from non-distinctive and commonly observed non-cancerous prostate pathological features, only four contained high-grade prostatic intraepithelial neoplasia; another five included BPH features. This small number precluded inclusion as independent parameters in the analyses.

### Statistical Analysis

The study divided cases into training and testing cohorts of similar patient populations, with analyses as follows. First, training cohort relative intensities of the analysed 36 spectral regions and the first 14 principal components (PC, representing cumulatively 80.4% total variance) were analysed using: (1) linear regressions against continuous variables (Vol% benign epithelia, cancer glands, and stroma; pre-surgical serum PSA; Free PSA%; and PSA density); (2) Analysis of variance (ANOVA, for normal distributions according to Shapiro-Wilk W tests), and/or Kruskal-Wallis-Wilcoxon test (for non-normal distributions) against categorical variables (PGG and pT); (3) Student’s t-test (for normal distributions with or without equal variance), and/or Mann-Whitney-Wilcoxon test (for non-normal distributions), for binary categorical variables (cancer vs. Hb, high vs. low aggressiveness, tumor recurrence vs. non-recurrence, and prostate cancer perineural invasion); and (4) Canonical analysis for discriminator (tumor recurrence vs. non-recurrence) discovery.

Statistical significance levels for the above two-sided tests were set at p < 0.0013 and p < 0.0035 for individual spectral regions and PCs, respectively, considering Bonferroni corrections. After Bonferroni corrections, each statistically significant spectral region, PC, or canonical score from the training cohort shown by statistical analysis to correlate with pathological or clinical parameters was: (a) systematically evaluated by analysis of covariance to eliminate the confounding potential of clinical factors, including patient age at surgery, pre-surgical serum PSA, and BPH status represented by prostate size (weight) (Table S2), and (b) compared with findings from samples in the testing-cohort to determine whether or not the correlation held. Results from analyses of covariance show that the statistically significant results discussed in the following sections were not impacted by these factors (Table S2).

For the testing-cohort, values of the PCs and canonical scores for each sample were calculated using the means, standard deviations, and coefficients (loading factors) obtained from the training-cohort. The two-sided statistical significance level for an individual test of a particular correlation (without multiple comparisons) for the testing cohort was set at p < 0.05. Unless otherwise indicated, only significant values (spectral regions, PCs, and canonical scores) proposed by the training cohort and verified by the testing-cohort through the above procedures are reported.

Finally, we evaluated statistical results measured from individual tissue samples of multiple specimens from single patients using mixed model analysis to ensure repetitive measurements did not confound reported statistical significance. Statistical analyses were performed by SAS-JMP (Cary, NC).

### Data availability

The datasets generated and analysed during the current study are available from the corresponding author upon request.

## Results

### Quantitative Histology

Quantitative H&E histological analysis of serial-sections for each entire specimen after MRS showed that only 27 (7%) samples contained cancer cells and glands, here termed “prostate cancer samples” (Training cohort: 20 samples, 5 patients; Testing cohort: 7 samples, 4 patients).

The remaining 338 samples were histologically-defined as Hb specimens from cancerous prostates (Training cohort: 179 samples, 77 patients; Testing cohort: 159 samples, 72 patients). This high Hb ratio agrees with observed low prostate cancer detection rates for random biopsy cores, which prompted changes in prostate cancer biopsy schemes from the classical sextant (6-core)^25^ to the current 12- to 24-core^26,27^ approach. Our MRS results unambiguously show a spectral power to identify prostate cancer specimens (Table S1), an ability seen in many studies by our group^11,28,29^ and others^24,30–37^; we thus do not concentrate here on this finding. Instead, we focused on the capacity of metabolomic information, measured from our large population of Hb specimens of cancerous prostates, to interrogate malignant status for prostate cancer diagnosis and patient prognostication. Due to the limited number of prostate cancer-positive, BPH, and Bx tissues, these groups are listed only as references, without analysis of intra-group differences.

### MRS differentiates PGG 1&2 from PGG 3&4

**Figure 2:**
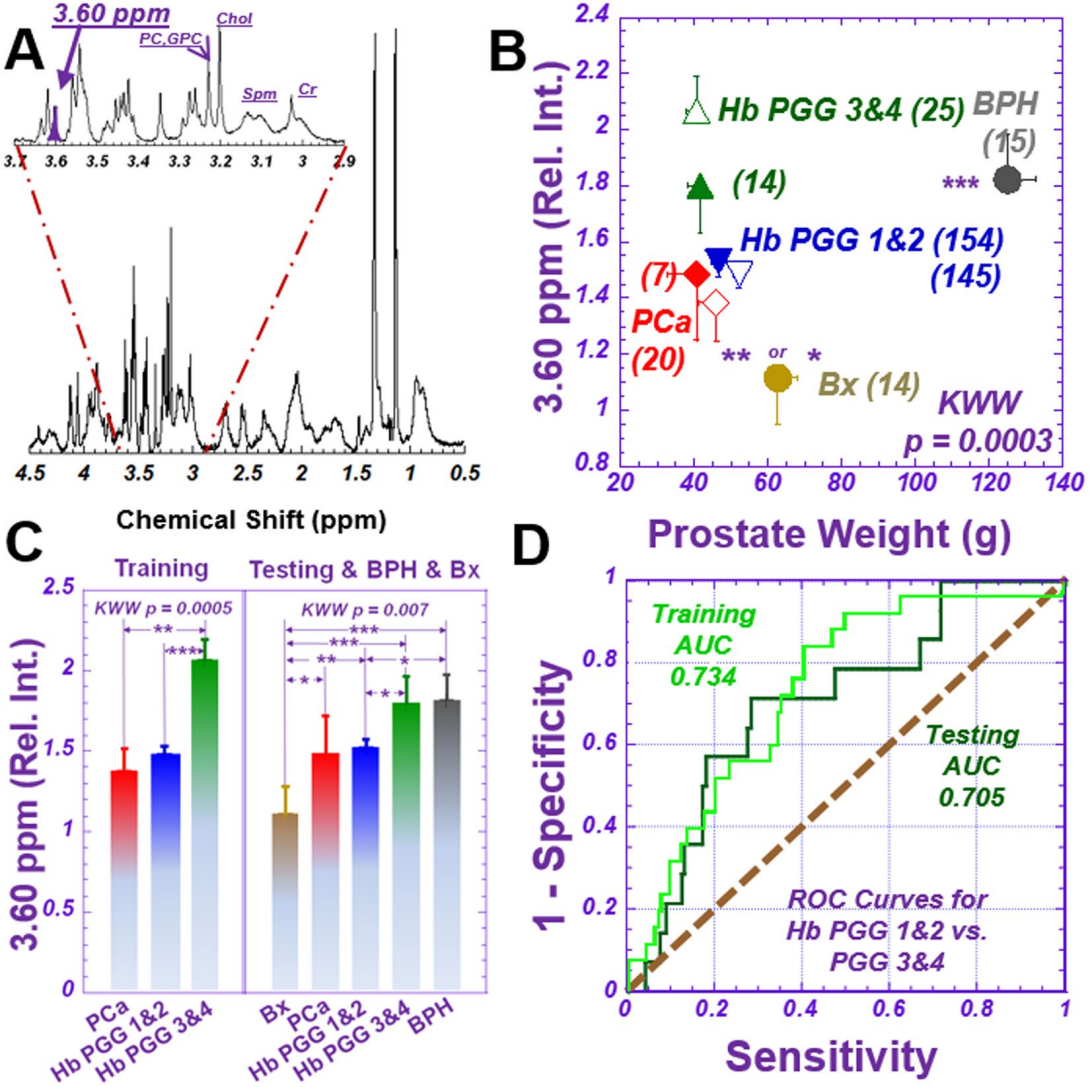
Differentiation of Prostate Cancer Prognostic Grade Groups (PGG). (**A**) A representative tissue proton MRS spectrum measured from a 10 mg tissue sample containing 49.7 (Vol%) cancer, 20.7 (Vol%) histologically-benign glands, and 30.0 (Vol%) stromal tissues. The sample was obtained from a 62-year-old patient of GS = 4 + 3 (PGG3) and pathological stage pT = IIc disease. The spectral region at 3.60 ppm (purple peak) represents the metabolites *m*-Ino, GPC, PC and Val, as well as other major metabolites. (**B**) Mean and standard error of relative spectral intensities at 3.60 ppm, according to prostate weight, for tissue samples of training (open symbols) and testing (solid) cohorts and of BPH (grey) and Bx (tan). Blue triangles represent Hb samples in PGG1&2; green triangles show PGG3&4. Prostate cancer samples are red diamonds. Numbers in parentheses denote group size. Significance symbols on BPH and Bx denote significance levels when these groups were compared with all others. (**C**) Comparisons of relative spectral intensities of the peak at 3.60 ppm among groups in the training and testing cohorts, respectively. Comparision details between PGG1&2 and 3&4 are: Training: *PGG1&2* Mean = 1.49 ± 0.06, Upper 95%CI = 1.60, Lower 95%CI = 1.37; *PGG3 & 4*: Mean = 2.07 ± 0.15, Upper 95%CI = 2.36, Lower 95%CI = 1.78, *p* < *0.0003*; Testing: *PGG1 & 2*: Mean = 1.53 ± 0.04, Upper 95%CI = 1.60, Lower 95%CI = 1.45; *PGG3 & 4*: Mean = 1.80 ± 0.12, Upper 95%CI = 2.04, Lower 95%CI = 1.56, *p* < *0.0114*), where Upper 95%CI and Lower 95%CI indicate upper and lower confidence intervals, respectively. (**D**) ROC curves presenting the overall accuracy of relative spectral intensities at 3.60 ppm for differentiating PGG1&2 from PGG3&4 groups for samples in the training and testing cohorts, respectively.

After analyzing the 36 spectral regions^18^ for Hb training cohort samples, we observed 7 regions that could differentiate between these two groups. Two of them (integrated peaks at 3.63 and 3.60 ppm) demonstrated significance after Bonferroni correction for multiple spectral-region comparisons of the 36 spectral regions. When analyzing these two significant regions for the testing cohort, we observed statistically significant increases in the 3.60 ppm spectral region (Fig. 2A) of Hb samples that corresponded with increases in patient PGG levels (Fig. 2B,C). In figures throughout the report, the significance levels are represented by: *, denoting two-sided statistical significance without Bonferroni correction (*p* < *0.05*); **, two-sided statistical significance without Bonferroni correction (*p* < *0.005*); and ***, two-sided statistical significance after Bonferroni correction (*p* < *0.0013*).

As tissue samples contain mixtures of all possible MRS-observable tissue metabolites, any spectral region (integrated peak) includes contributions from many known or yet-to-be-defined metabolites. Thus, instead of presenting results as individual metabolites, we present all statistically significant correlations in terms of spectral regions and list their major, possibly contributing, metabolites (Table S1). For instance, the 3.60 ppm region may include the metabolites *myo*-Inositol (*m*-Ino), glycerophosphocholine (GPC), phosphocholine (PChol), and valine (Val) (purple label, Fig. 2A). This 3.60 ppm spectral region can differentiate, with statistical significance, between the PGG1&2 and PGG3&4 groups for each cohort, at an overall accuracy of 73% and 71% (Fig. 2D) for the training and testing cohorts, respectively, and for the entire studied population (*p* < *0.0001*).

### MRS differentiates pT = IIab from pT = IIc Groups

**Figure 3:**
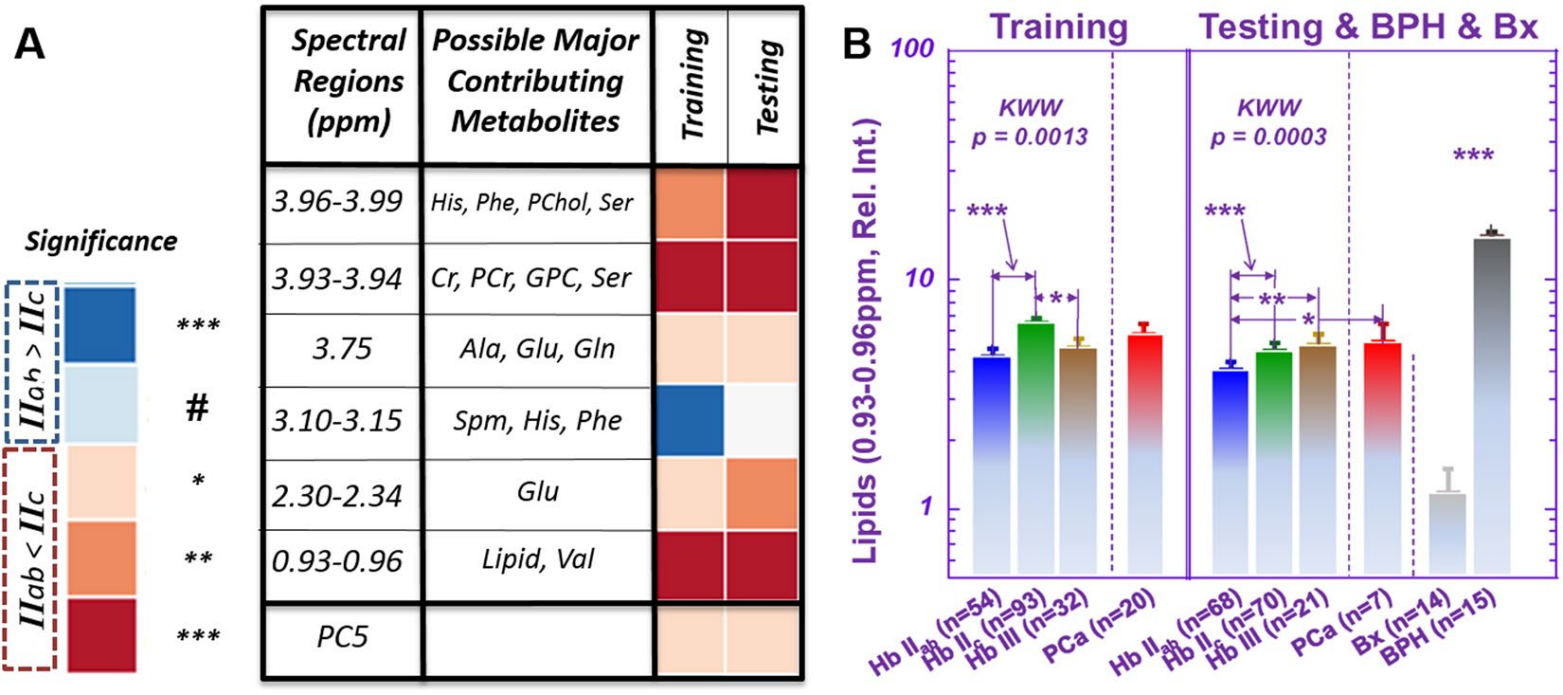
Differentiation of pathological stages. Signifiance levels (***, **, and *) are defined in the text, whereas the ‘#’ notation indicates a one-sided test with p < 0.05. **(A)** Multiple spectral regions showed a potential to differentiate pT = IIab from pT = IIc groups. **(B)** For one of these regions, (0.93–0.96), Kruskal-Wallis-Wilcoxon (KWW) results are also shown for comparing pT = IIab, pT = IIc, and pT = III groups. The major contributing metabolites to the relative spectral intensities at 0.93–0.96 ppm are mostly free lipids, with very limited intensity from Val. Means and standard errors are plotted in the figure. The notation ‘***’ above the BPH and Bx groups indicates that both groups are significantly different from all other groups. The comparision details between IIab and IIc are: Training: *IIab*: Mean = 4.66 ± 0.40, Upper 95%CI = 5.45, Lower 95%CI = 3.88; *IIc*: Mean = 6.48 ± 0.31, Upper 95%CI = 7.09, Lower 95%CI = 5.88, *p* < *0.0013*; Testing: *IIab*: Mean = 4.07 ± 0.17, Upper 95%CI = 4.41, Lower 95%CI = 3.73; *IIc*: Mean = 4.98 ± 0.17, Upper 95%CI = 5.31, Lower 95%CI = 4.65, *p* < *0.0003*.

PGG evaluates cellular morphological alterations seen in cancer cells and glands, considered clinically significant due to their correlation with patient prognostication. PGG may initially be assessed at biopsy, but determining prostate cancer pathological tumor stage (pT), can be accomplished only after radical prostatectomy with histology evaluation of the whole prostate. Nevertheless, our Hb sample MRS results show a significant ability for multiple spectral regions to differentiate pT = IIab from pT = IIc groups (Fig. 3A). Among these spectral regions, the 0.93–0.96 ppm (primarily MRS-visible lipid droplets) region can differentiate pT = IIab from pT = IIc groups in the training (*p* < *0.0013*) and testing (*p* < *0.0004*) cohorts, respectively (Fig. 3B). As pT = II denotes prostate cancer confined to the prostate, IIab to less than half the prostate, and IIc throughout the prostate, this differentiation likely reflects metabolic alterations due to tumor size. However, this relationship to tumor size does *not* extend to pT = III, where prostate cancer extends beyond the prostate capsules, introducing myriad factors affecting metabolism.

At present, pT can only be determined after surgical removal of the prostate. Thus our findings, achieved by metabolic measurement of small tissue samples (~mg in weight and comparable to a biopsy core), support the potential clinical usefulness of measurements that can be obtained by using biopsy cores to help inform pre-treatment decision-making by assessing prostate cancer pT stage before radical prostatectomy.

### MRS distinguishes cancer aggressiveness

Based on pathologically identified cancer aggressiveness after radical prostatectomy, we divided the PGG1&2&3 cases of each cohort into two groups. The less aggressive group included cases of PGG1&2 and pT = II; the more aggressive group, either PGG3 or pT = III. PGG4&5 cases were excluded from analysis owing to their obvious aggressiveness.

**Figure 4:**
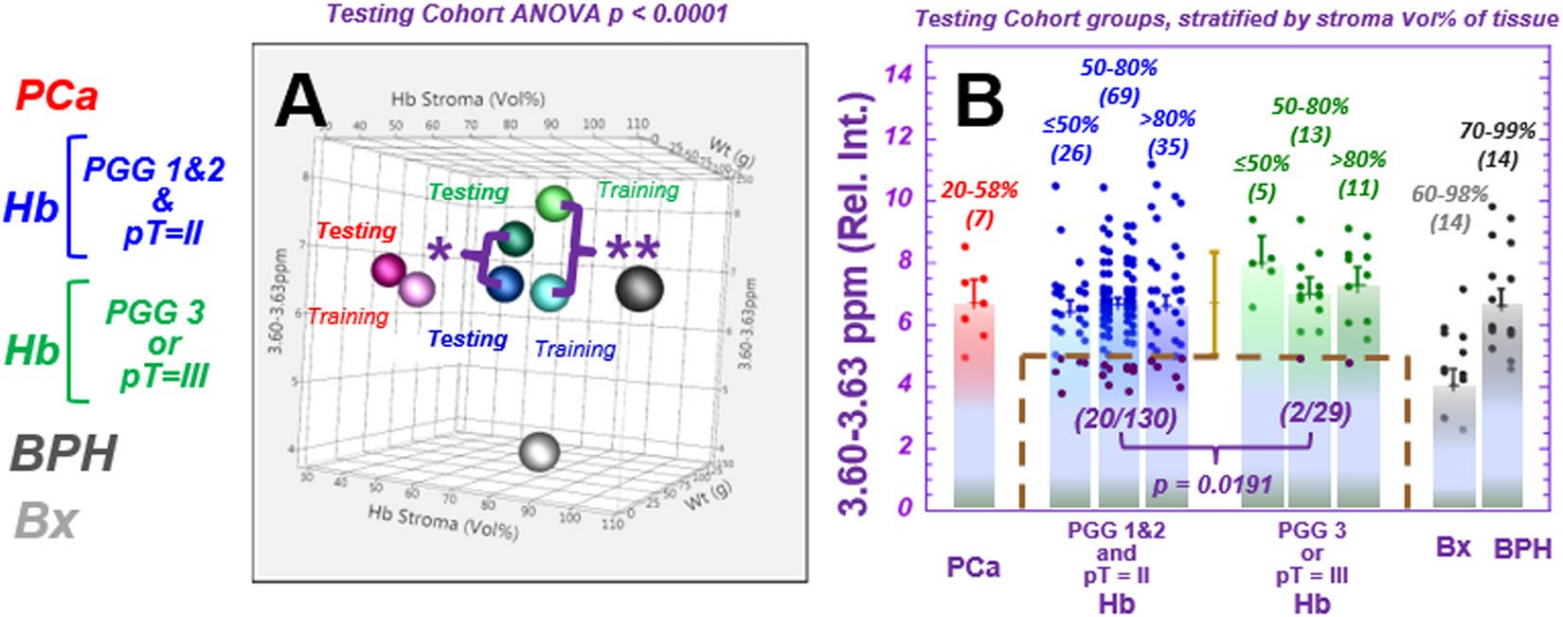
Spectral region 3.60–3.63 ppm shows distinguishing power between low and high aggressive cases. The patient population was divided into (1) Low aggressiveness cases: PGG1&2 and pT = II (Blue, 58 cases for both the training and testing cohorts, and 139 and 130 individual samples for each cohort, respectively); and (2) High aggressiveness cases: PGG3 or pT = III (green, 13 and 14 cases for each cohort, respectively, and 29 individual samples for each cohort). (**A**) A 3D illustration of the mean values of relative spectral intensities at 3.60–3.63 ppm (*m*-Ino) of samples in the training and testing cohorts vs. stroma composition (Vol%) in the tissue and prostate weight. Prostate cancer samples (red), Hb groups organized according to High (green) and Low (blue) aggressiveness, BPH (dark grey) and Bx (silver) are shown. The training and testing cohorts are represented by light and dark symbols, respectively. Significant differences (ANOVA *p* < *0.0001*) are observed among groups in the testing cohort (including BPH and Bx groups) and for all groups (Hb, High 7.54 ± 0.27; Hb, Low 6.55 ± 0.12; prostate cancer 6.50 ± 0.39; BPH 6.64 ± 0.52; and Bx 4.04 ± 0.53). (**B**) For samples stratified by Vol% stroma, a Kruskal-Wallis-Wilcoxon comparison of relative spectral intensities at 3.60–3.63 ppm from Hb samples shows statistical significance in differentiating Low from High aggressive groups for the testing cohort. Furthermore, this region could identify a subgroup (20/130) of samples in the Low aggressive group (magenta points in the brown dashed box), using a threshold of one standard deviation below the mean calculated for all Hb testing cohort samples. This differentiation capability is independent from that of tissue stroma composition. Values above parentheses represent the amount (vol%) of stroma in the samples, and numbers in parentheses denote the number of samples in the group. The comparision details between High and Low are: Training: *High*: Mean = 7.81 ± 0.43, Upper 95%CI = 8.66, Lower 95%CI = 6.96; *Low*: Mean = 6.50 ± 0.20, Upper 95%CI = 6.89, Lower 95%CI = 6.11, *p* < *0.0026*; Testing: *High*: Mean = 7.27 ± 0.31, Upper 95%CI = 7.88, Lower 95%CI = 6.66; *Low*: Mean = 6.60 ± 0.15, Upper 95%CI = 6.88, Lower 95%CI = 6.31, *p* < *0.0191*.

When examining spectral regions able to differentiate less vs. more aggressive prostate cancer, we observed a significant distinguishing power in the 3.60 ppm spectral region for both training (*p* < *0.0017*) and testing (*p* < *0.0409*) cohorts. As the resonance 3.60 ppm includes one of the triplets from *m*-Ino, we included the other *m*-Ino peak and analysed the 3.60–3.63 ppm region. We further calculated the power of the 3.60–3.63 ppm spectral region to differentiate between the two groups; this yielded statistically significant separations for the training (*p* < *0.0026*) and testing (*p* < *0.0191*) cohorts for individual tissue samples (Fig. 4A), independent of tissue histology composition. Along with differentiating low or high malignancy potential, this spectral region could identify a prostate cancer sub-group of the low malignant potential group in both the training and testing cohorts, with the latter shown by purple dots in the brown boxed area in Fig. 4B. This group was identified by a threshold of one standard deviation below the mean of Hb samples in the testing cohort, and the separation was shown to be independent of tissue stroma composition.

**Figure 5:**
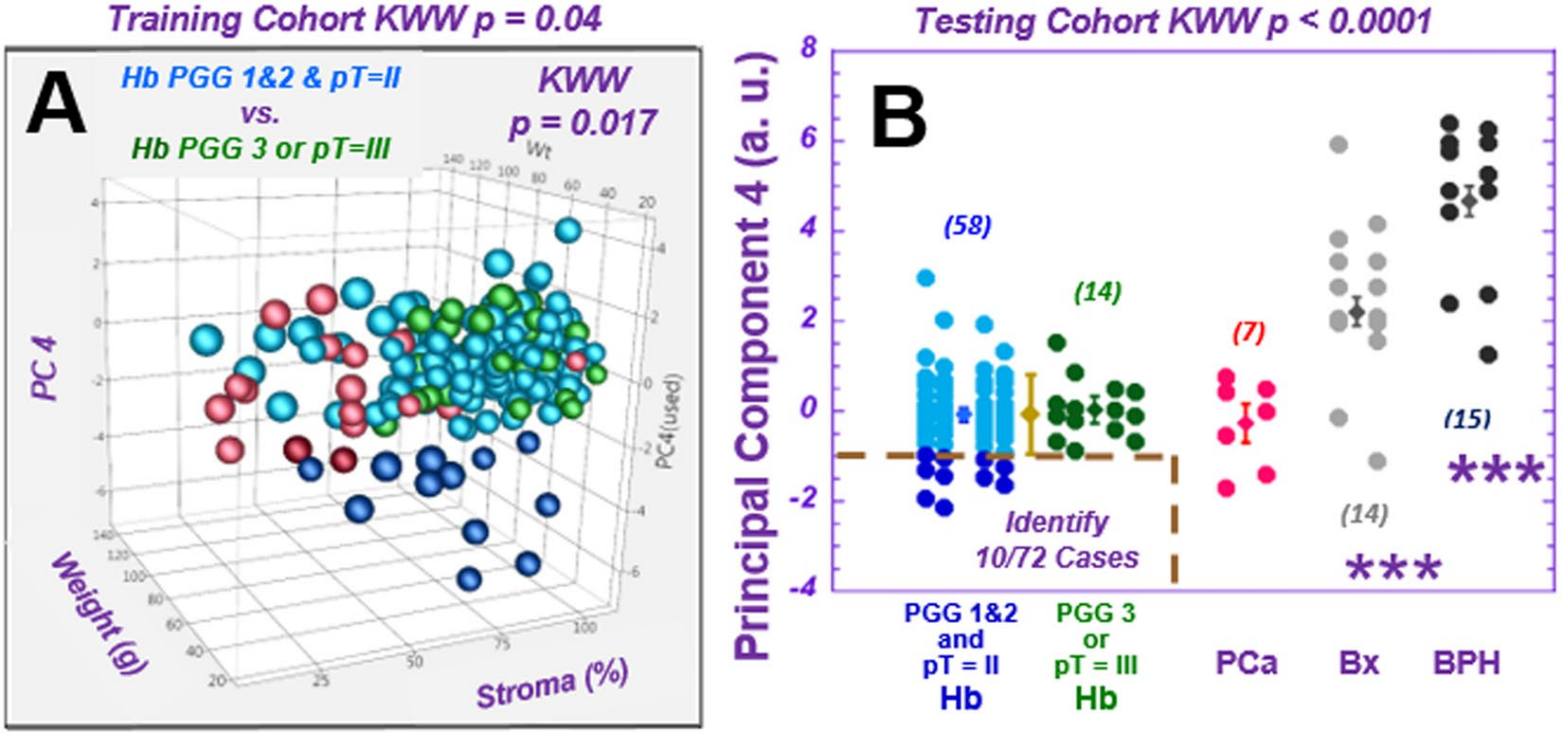
Identification of a sub-group of prostate cancer cases with low aggressive potential. (**A**) A 3D illustration of the mean values of principal component (PC) 4, as calculated from individual Hb samples vs. prostate weight and the percentage of stroma in samples for the training cohort. The subgroup of samples in the Low aggressive group, identified by a threshold of one standard deviation below the mean (M-SD), is highlighted by dark blue points. (**B**) The values of PC4 calculated for the testing cohort according to parameters obtained from the training cohort can identify 10 from 58 (17%) Low aggressive cases, using the threshold of M-SD measured from Hb samples (below the brown dashed line). Prostate cancer, BPH, and Bx values are also presented for references.

Results of a similar trend (the values for more aggressive cases are higher than for less aggressive ones) were observed with principal component (PC) 4, calculated from all spectral regions of the training cohort. PC4 differentiates the two malignancy potential groups for the training group (*p* < *0.0017*) by identifying a subgroup of the low malignant potential cases one standard deviation below the mean (dark blue points) (Fig. 5A**)**. When applying the training cohort’s PC4 calculating coefficients (Table S3) to cases in the testing-cohort, the result identifies 10 (dark blue points, Fig. 5B) from 58 (17%) of the low aggressive cases among all 72 (14%) cases in the testing-cohort. Five of these ten cases were also identified in the testing cohort by spectral region 3.60–3.63 ppm.

The loading coefficients for PC4 (Table S3) agree with results in Fig. 4, as we observed major positive loading coefficient contributions from *m*-Ino. In addition, major positive contributions from glutamate (Glu), and negative contributions from lactate (Lac), choline (Cho), glutathione (GSH), and pyruvate (Pyr) were also observed for PC4 calculated from the Hb tissue of the training cohort.

Of great interest, the mean value of the 3.60–3.63 ppm data for cancer-containing samples (6.50 ± 0.39) was observed to be similar to that of Hb samples in the low aggressive group (6.55 ± 0.12) (Fig. 4B). Both were significantly lower (*p* < *0.0026*) than the mean value of Hb samples in the high aggressive group (7.54 ± 0.26). Although this spectral region represents myo-inositol (*m*-Ino) and other metabolites, including GPC, PChol and Val, our analyses did not show measurable GPC, PChol and Val changes in other spectral regions. The differences observed may specifically indicate an increase in *m*-Ino in the Hb samples adjacent to cancer of the high aggressive group.

### MRS assists in predicting cancer recurrence

Pathology of the entire prostate serves as the gold standard for all metabolomic profile results presented thus far; therefore, all findings could have been achieved by current histology techniques after radical prostatectomy. Reducing overtreatment, however, motivated this study. Furthermore, even given histological evaluation of the entire prostate gland after radical prostatectomy, serious challenges still beset the prostate cancer clinic, particularly for (1) predicting tumor biochemical recurrence (BCR), defined as elevation of blood serum PSA levels after prostatectomy as a surrogate marker of prostate cancer progression^20^, and (2) evaluating patient overall survival. The challenges of predicting recurrence and evaluating overall survival are particularly troublesome for cases deemed to be less aggressive (e.g. PGG 2 and pT = II).

Among the 158 prostatectomy cases studied, 39 cases (18 training, 21 testing cohort) presented BCR in >15 years of follow-up. Among them, we found ten BCR cases of PGG 1&2&3 and pT = II for each cohort. Time gaps between prostatectomy and recurrence for the training and testing cohorts were 56 ± 36 (Max 104, Min 9) and 68 ± 29 (129, 10) months, respectively. Each BCR case was multivariably-paired according to tissue pathology features, PGG, PSA density, and age at prostatectomy (Table S4) as closely as possible with a BCR-free case from the same cohort. The BCR-free cases were also selected for longer follow-up time (Training: 128 ± 29, and Testing: 140 ± 28 months) to ensure BCR-free status. Of note, this matching process rendered ineffective any currently known nomograms^38,39^ for predicting BCR potential.

**Figure 6:**
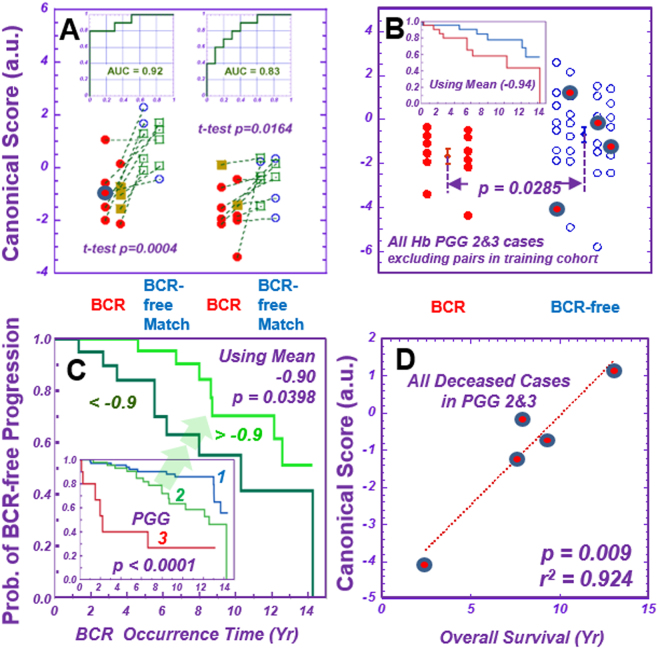
Prediction of prostate cancer potential at the time of surgery. Metabolomics measured from Hb tissues produced profiles able to differentiate prostate cancer biochemical recurrence (“BCR” vs. “BCR-free Match”). (**A**) Ten BCR cases of PGG 1&2&3, all pT = II, were identified from the training and testing cohorts, respectively, and paired with BCR-free Match cases from the same cohort. Metabolomic profiles differentiating BCR from BCR-free Match cases were calculated with the training cohort, applied to the testing cohort, and yielded significant results. The ROC curves with overall accuracies for both the training and testing cohorts are shown as insets in the figure. See text for details of canonical score analysis. (**B**) The application of discriminant canonical score calculations onto PGG2&3 cases for the two cohorts combined yields a general, significant differentiation between BCR and BCR-free cases, as well as two distinct Kaplan-Meier estimator curves that emerge according to when BCR occurred after prostatectomies (inset) for the studied population. (**C**) Our studied prostate cancer population agrees with the published overall BCR Kaplan-Meier estimator curves for PGG 1, 2, and 3 cases^20^. However, our BCR metabolomic discriminant canonical values were able to significantly subdivide cases of the single, clinicopathologically indivisible PGG2 group into two distinct Kaplan-Meier estimator groups for prostate cancer recurrence by using the mean value (−0.9) as a threshold. (**D**) Canonical score values correlate with patient overall survival times for all five deceased patients within the analyzed 65 PGG2 and 3 cases. These five cases are also blue-circle labelled in Fig. 6A,B.

Analyzing spectroscopic data from prostatectomy samples obtained >15 years ago allowed us to investigate metabolomic profiles that might differentiate patients of BCR versus BCR-free status. Canonical analyses, performed in the training cohort on principal components (PC 2, 3, 7, 8, and 10) calculated from 36 spectral regions of ten pairs of BCR and BCR-free cases, produced a discriminating score that differentiated BCR from BCR-free in the training-cohort (Fig. 6A). These five PCs were selected as they yielded the smallest p-values, ranging from 0.11 to 0.25, according to Student t-tests conducted on BCR and BCR-free groups of the training cohort. We applied all parameters of the training-cohort, including overall coefficients for each spectral region (Table S5), to the corresponding spectral regions of the testing cohort to evaluate this discriminating score’s efficacy for the testing cohort.

The calculated score values were able to significantly differentiate BCR and BCR-free cases in the testing-cohort with an overall accuracy of 83%, seen in the receiver operating characteristic (ROC) curves in Fig. 6A. The major contributing metabolites for BCR included Cho, PChol, GPC, and Glu, but for BCR-free, *m*-Ino.

To test canonical scores against matched cases in the testing cohort, we applied canonical score calculations to PGG2&3 cases for both the training and testing cohorts. We excluded paired cases in the training cohort used to develop the formula and were cognizant that the discriminant scores obtained from BCR and BCR-free matched cases in the training cohort were heavily skewed towards PGG2&3 cases. We observed a statistically significant general differentiation between BCR and BCR-free cases (Fig. 6B). We then set the mean of calculated score values from these cases as a threshold: values above and below this mean clearly produced two distinct Kaplan-Meier estimator curves of time of BCR occurrence after prostatectomy (Fig. 6B, inset).

Overall BCR Kaplan-Meier estimator curves for our PGG1&2&3 groups present significant inter-group differentiations and agree with published data^20^. These BCR discriminant scores also provide statistically significant intra-group differentiation for PGG2 cases, as seen in Fig. 6C. We tested the potential of this score to predict patient overall survival, and observed a significant linear relationship between score values and survival times for all five deceased patients in our 65 analyzed PGG2&3 cases (Fig. 6D).

After observing the significant potential of metabolomics analyses to discriminate BCR status for the PGG2 group, we tested a number of other currently known clinical parameters, including pre-surgical PSA (mean 5.94 ng/mL), pT stage (II vs. III), and prostate cancer perineural invasion (+ vs. −), to assess their potential to provide further discrimination within the PGG2 group. None of these parameters, alone or in combination, proved capable. However, when the metabolomic parameter in Fig. 6C was combined with the patient pre-surgical PSA value, discriminating power increased from p < 0.040 (metabolomics alone) to p < 0.003 (combination value).

## Discussion

MRS seeks to address the clinical challenges of prostate cancer diagnosis and characterization by measuring disease-related metabolic alterations. Metabolites localized on cancer lesions are expected to aid cancer detection by their differentiation from Hb surroundings. A large body of medical research and clinical imaging studies support this assumption. Using histology of an entire removed prostate as the gold standard, we instead investigated the assumption that cancer-related metabolic alterations precede morphological changes.

The phenomenon of a cancer metabolite field-effect, which we imply and which has been suggested in prior studies^11,40^, coincides with proposed tumor-associated-stroma effects, through which cancer growth in adjacent epithelium occurs by transforming the biology of adjacent Hb epithelium into an environment with cancer characteristics^41,42^. Along this line of research, published results have reported cell signalling between benign and cancer cells^14,17^, transfer of genomic information^13,15^, or secreted proteins or receptor expression^12,16^ which enable or are necessary for tumor growth. Metabolomics builds on these alterations and presents targeted, functional read-outs of these upstream tumor biological changes to further demonstrate evidence of a cancer field effect. Studies comparing the metabolomic profiles of histologically normal tissue adjacent to esophageal cancer^43^ or adjacent to precancerous colorectal neoplasms^44^ support the theory of metabolomic cancer field effects. We extend this theory by presenting, for the first time, MRS metabolomic profiles of Hb specimens from cancerous prostates which can achieve the following: (1) differentiate PGG1&2 from PGG3&4, (2) differentiate pT = IIab from pT = IIc groups, and (3) identify a sub-group of the low aggressive prostate cancer group. Additionally, our MRS-based metabolomics (4) showed elevated *myo*-inositol levels, an endogenous tumor suppressor and potential mechanistic therapy target, in patients with highly-aggressive cancer and (5) indicated the ability to predict  prostate cancer biochemical recurrence and survival time.

The phenomenon of cancer field effects allows metabolomic profiles measured from Hb tissue to identify prostate cancer and predict disease aggressiveness, an ability that we account for in two ways. First, it may proceed from cancer *metabolomic fields*, through which MRS-visible metabolites of low molecular weight residing unbound in cytoplasm migrate from histology-defined cancer lesions into surrounding Hb structures. Second, it may represent the presence of *metabolomic lesions*, wherein advanced metabolic changes are detectable prior to formation of the cellular, structural abnormalities seen in histology. The idea of a cancer metabolomic ‘lesion’ having an impact beyond its histological foci can help overcome acknowledged clinical limits now imposed by histological sampling errors. It leads to the possibility that MRS measurement of tissue could identify suspicious areas missed by a biopsy needle, as well as supplement or yield disease information independent from that of morphology-based histology, upon which we elaborate below.

This retrospective study has a number of intrinsic limitations. For prostatectomy specimens, we cannot retrieve information on the distance between Hb tissues analysed and prostate cancer foci; thus we cannot quantify the scale of observed metabolomic fields or lesions. However, our statistically significant results, obtained from rigorous experimental design (training and testing cohorts of similar populations) and data analyses (Bonferroni corrections, covariance analysis, mixed model analysis, etc.), are extremely unlikely to result from random coincidence. Thus, the significant correlations observed, despite the possible confounding variable of unknown distance, strongly suggest that future studies incorporating distance will enhance correlations.

No healthy human prostate tissue, absent clinical suspicion, may be removed. Thus the *ex vivo* nature of our study and our use of mostly diseased samples, collected from prostate cancer-positive or BPH prostates, or from patients with elevated PSA values, must be acknowledged. Other sources, such as histologically-proven, cancer-free prostate tissues from such surgical procedures as cystectomy (removal of a cancerous bladder) or autopsy also present intrinsic uncertainties. Known or unknown metabolic effects may have occurred in these prostate tissues of cystectomy patients, despite their histologically healthy appearance. Samples obtained through autopsy may be unsuitable for metabolomic analysis due to gaps between subject death and tissue preservation. Such clinical difficulties may be circumvented by clever systematic designs, such as the use of prostate biopsy samples from subjects for whom elevated PSA findings warrant biopsy, but who thereafter present with no prostate cancer for a number of years, as demonstrated in this study. By further establishing prostate cancer metabolomics from such ‘conditional controls’, studies can contribute to the urgent need for markers in the prostate cancer clinic, while operating in accordance with accepted ethical and empirical principles of medical science and clinical practice.

Our results agree with recent widely reported and reviewed knowledge of cellular metabolism in prostate cancer and other malignancies’ development and progression^45^; we will thus elaborate on only a few new insights.

High concentrations of Spm, a well-known metabolite in normal human prostates^23,24,29^, with reductions during prostate cancer development and progression, form the basis of MRS imaging detection of prostate cancer. Published reports state that Spm differentiates prostate cancer from adjacent adjacent Hb samples with statistical significance^37^, but our measurement of 27 prostate cancer and 338 Hb samples did not agree (Table S1). However, by further dividing Hb groups according to tissue epithelial composition, Spm was able to significantly differentiate prostate cancer-containing samples from Hb samples with high epithelial volume percentage (>40%) (*p* < *0.0007*). This result cautions against an overly generalized use of tissue metabolites as prostate cancer biomarkers.

Our most interesting metabolic finding suggests an increase in the intensities of *m*-Ino in the Hb tissue of high aggressive diseases, as compared with Hb tissue from low aggressive prostate cancer and cancerous prostate tissue samples. *M*-Ino, a six-fold ring sugar alcohol molecule, is involved in a number of biological processes, including osmotic adjustments and protection to balance cellular osmotic pressure, in cell signaling and second messenger systems, and by signal transmission for various hormones, neurotransmitters, and growth factors. Documented abnormalities in *m*-Ino metabolism are implicated in various disease states, including diabetes, renal disorders, and cancer^46^. Of note, *m*-Ino anticancer activity is reported to induce apoptotic cell death, arrest cancer cell cycle and cell line differentiation in prostate^47^, and inhibit lung carcinogenesis^48,49^.

As *m*-Ino is a major MRS measurable metabolite, several analyses of the metabolite in intact human prostate tissues^30,36,37^ and human prostatic fluid^23,24^ are reported. These studies analyzed Hb samples as a single group, without assessing *m*-Ino differences in Hb tissues in groups of different aggressiveness. Considering *m*-Ino’s association with oncogenic PI3K pathways and as a proposed cancer inhibitor in human lung cancer^48^, our *m*-Ino results may indicate its function as an Hb tissue defense response in cancerous prostates, one providing endogenous tumor suppression of aggressive prostate cancer growth.

Using metabolomic profiles validated with a testing cohort, we present cancer metabolomic thresholds to assist diagnosis and prognosis in ways which are beyond the scope of existing prostate cancer clinical and pathological criteria. Successful application of metabolomic thresholds is unlikely to yield a crisp diagnosis or prognosis for all patients, but threshold sub-group identification could immensely impact decision-making and quality of life. For a disease of high incidence, such as prostate cancer, the ability to confidently identify low aggressive status – even for the 10% sub-group of prostate cancer patients shown in Fig. 5 – could allow more personalized medicine worldwide for over 110,000 prostate cancer patients annually and 16,000 men in the USA alone^5,50^.

In addition, metabolomics findings subdivided the single, clinical PGG2 group into *two distinct groups* of statistically significant difference in their Kaplan-Meier biochemical recurrence curves. By differentiating these distinct PGG2 cases, currently each considered to represent low-aggressive prostate cancer, clinicians could be alerted, after a patient’s biopsy but before prostatectomy, to more closely monitor those who show an increased potential for recurrence. Further validation of these results through multicenter trials can lead to clinical translation of these *ex vivo* results and to *in vivo* clinical MRS metabolomics protocols to aid decisions on prostate cancer treatment.

The metabolomic conceptual advance demonstrated here for diagnosing and characterizing human cancer by molecular evaluation of Hb specimens can dramatically transform the current, morphology-based histology paradigm. Complementing it with a metabolomics-informed evaluation system that is sensitive to cancer biology in individuals will advance the realization of personalized medicine and enable more informed clinical decision-making.

## Electronic supplementary material

Supplementary Information
