# Supplementary material for: Metabolomic Prediction of Human Prostate Cancer Aggressiveness: Magnetic Resonance Spectroscopy of Histologically Benign Tissue
Source: Sci Rep. 2018 Mar 26;8:4997. doi: 10.1038/s41598-018-23177-w (PMC5980000; doi:10.1038/s41598-018-23177-w)
Supplement: Supplementary file 1 — Supplementary Information [file 41598_2018_23177_MOESM1_ESM.pdf]

Metabolomic Prediction of Human Prostate Cancer Aggressiveness: Magnetic Resonance Spectroscopy of Histologically Benign Tissue

Lindsey A. Vandergrift[1], Emily A. Decelle[1], Johannes Kurth[1,4], Shulin Wu[1], Taylor L. Fuss[1],

Elita M. DeFeo[1], Elkan F. Halpern[2], Matthias Taupitz[4],

W. Scott McDougal[1,3], Aria F. Olumi[1,3], Chin-Lee Wu[1*], and Leo L. Cheng[1,2*]

[1]Department of Pathology, Massachusetts General Hospital, Harvard Medical School,

Boston, Massachusetts, 02114 USA

[2]Department of Radiology, Massachusetts General Hospital, Harvard Medical School,

Boston, Massachusetts, 02114 USA

[3]Department of Urology, Massachusetts General Hospital, Harvard Medical School,

Boston, Massachusetts, 02114 USA

[4]Department of Radiology, Charité Medical University of Berlin, Chariteplatz 1,

10117 Berlin, Germany

Supplementary Information

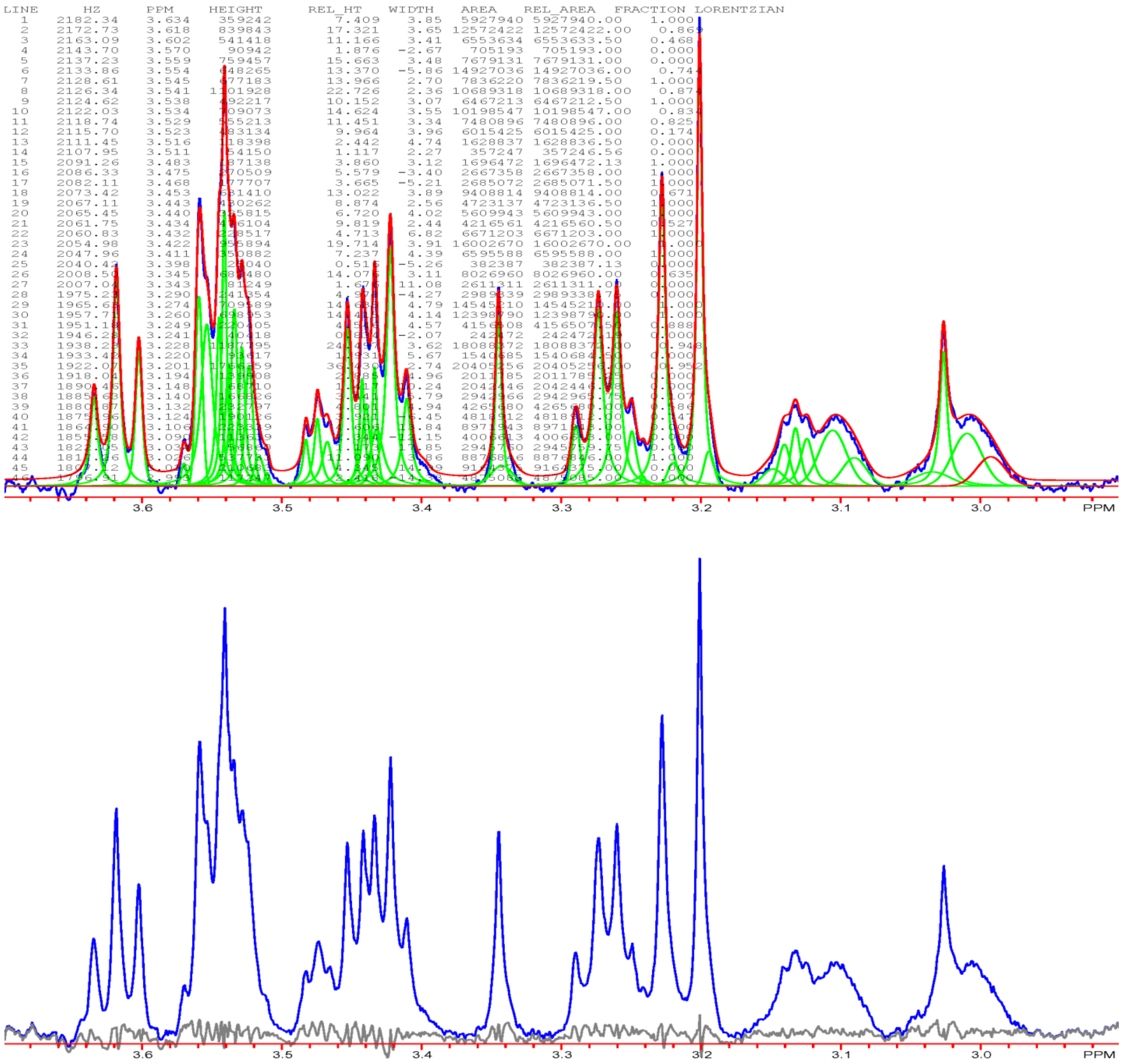

Fig S1. An example of a curve fitted spectral region. The top panel presents the results of Lorentzian-Gaussian curve fitting achieved in the spectral region (2.9-3.7 ppm). Green curves represent individual fitted peaks (with fitting parameters listed in the figure), while the red curve (except the far right individual red peak at 2.90 ppm) represents the sum of individual curves that overlay with blue experimental curves. The bottom panel presents the experimental curve in blue and differences between the experimental and fitted curves in grey.

Table S1. Linear correlations (*r* values) of spectral region relative intensities with volume percentages of prostate cancer pathologies quantified from tissues after MRS, and Student t-test on prostate cancer (PCa) (n=27) and Hb (n=338) groups. (* possible major contributing metabolites were listed according to chemical shift values seen in literature; ** indicate p<0.05 after Bonferroni corrections).

Res.	Major Metabol*.	HB Epi (Vol%)	Prostate cancer (Vol%)	Stroma (Vol%)	Prostate cancer vs. Hb
**p414-410**	*Lac*	**, -0.168	**, +0.168		**, PCa>Hb
**p405**	*m-Ino*	**, 0.182			
**P399-396**	*His, Phe, PC, Ser*				
**p394-393**	*Cr, PCr, GPC, Ser*	**, -0.181		**, 0.175	
**P378**	*Ala, Glu, Gln, GSH*				
**P375**	*Ala, Glu, Gln*		**, 0.174		
**P374-371**	*Ala, Glu, Gln*	**, -0.328		**, 0.204	
**p363**	*m-Ino, GPC, PC*				
**p362**	*m-Ino, GPC, PC*	**, 0.194			
**p36**	*m-Ino, GPC, PC, Val*				
**p334**	*s-Ino*				
**p329**	*m-Ino, Tau*				**, PCa<Hb
**p327**	*m-Ino, Tau, Phe*		**, -0.171		**, PCa<Hb
**p326**	*m-Ino, Tau*	**, -0.175		**, 0.245	**, PCa<Hb
**p324**	*m-Ino, Tau*	**, -0.176		**, 0.250	**, PCa<Hb
**p322**	*PC, GPC, His*		**, 0.178		**, PCa>Hb
**p320**	*Cho*	**, -0.211			
**p315-310**	*Spm, His, Phe*	**, 0.428		**, -0.363	
**p309-305**	*Spm*	**, 0.273		**, -0.235	
**p303**	*Cr, PCr*				
**p271**	*Cit*	**, 0.487		**, -0.435	
**p268**	*Cit*	**, 0.368		**, -0.354	
**p255**	*Cit, GSH*	**, 0.391		**, -0.333	
**p252**	*Cit, GSH*	**, 0.299		**, -0.256	
**P245-p243**	*Gln*	**, -0.174		**, 0.171	
**P242-p240**	*Gln*				
**P236**	*Glu, Pyruvate*				
**P235**	*Glu*		**, 0.216		**, PCa>Hb
**P234**	*Glu*				
**P233-p230**	*Glu*				
**p147**	*Ala*				
**p133**	*Lac, (Lip)*	**, -0.214		**, 0.204	
**p128**	*Lip*				
**P120**					
**p096-p093**	*Val, Lip*	**, -0.262		**, 0.218	
**p090**	*Lip*	**, -0.175			

Abbreviations: Res, resonance region; Lac, lactate; m-Ino, myo-inositol; Cr, creatine; PCr, phosphocreatine; GPC, glycerophosphocholine; Ala, alanine; Glu, glutamate; Gln, glutamine; GSH, glutathione; His, histidine; Phe, Phenylalanine; PC, phosphocholine; Val, valine; s-Ino, scyllo-inositol; Tau, taurine; Cho, choline; Ser, serine; Spm, spermine; Cit, citrate; Lip, lipids. “p414-410” indicates spectral region 4.14-4.10 ppm, “PCa>Hb, or PCa<Hb” indicating the mean values of the relative intensity of the specific spectral region measured for prostate cancer group are greater, or smaller, than that measured for the Hb group.

Table S2. Evaluations of prostate cancer metabolites and metabolomic profiles reported against patient age at surgery, pre-surgical serum PSA and BPH status in terms of prostate size by general linear model of co-variance analysis. Results below indicate the preservation of the statistical significances of the reported prostate cancer metabolites and metabolomic profiles in differentiating prostate cancer aggressiveness parameters after consideration of these clinical factors as co-variances. (e.g. The 3.60 ppm resonance in Figure 2B differentiates among all groups in the training and testing cohorts, including BPH and Bx groups, after inclusion of "age" as a co-variant, and the resulting differentiating significance is p<0.0001 shown in the "Groups Prob>F" column.) Table S2 should only be used in conjunction with the results presented in Figures 2, 3, and 4 to indicate the statistical significances presented in those figures are free from the interference of the three clinical factors examined.

Co-variances Tested	Number of Parameters	Degrees of Freedom	Sum of Squares	F Ratio	Groups Prob > F
Figure 2B. 3.60ppm Between testing cohort Hb PGG=1&2 and PGG=3&4 Groups					
**Age**	1	1	0.9527	4.5414	***0.0347***
**Pre-Surg PSA**	1	1	1.0158	4.9922	***0.0269***
**Wt(gm)**	1	1	0.9663	4.6095	***0.0333***
Figure 3B. PC4 Groups in Testing Cohort including BPH and Bx					
**Age**	4	4	236.5749	43.8779	***<0.0001***
**Pre- Surg PSA**	4	4	279.2127	51.6032	***<0.0001***
**Wt(gm)**	4	4	101.227	19.3569	***<0.0001***
Figure 4A. Canonical Score Between testing cohort BCR and non-BCR match groups					
**Age**	1	1	5.66432	6.5986	***0.0199***
**Pre- Surg PSA**	1	1	5.214275	6.2249	***0.0232***
**Wt(gm)**	1	1	5.368418	6.4232	***0.0214***

Table S3. The coefficients for each spectral region in calculating PC4 in Figure 3.

Spectral Regions	Coefficient
p414-410	***-0.453***
p405	-0.108
p399-396	0.107
p394-393	0.205
P378	0.097
P375	0.202
P374-371	0.077
p363	***0.236***
p362	***0.278***
p36	***0.305***
p334	0.144
p329	-0.004
p327	-0.039
p326	0.006
p324	0.128
p322	0.026
p320	***-0.163***
p315-310	-0.008
p309-305	0.071
p303	0.076
p271	-0.106
p268	-0.096
p255	***-0.131***
p252	-0.004
P245-p243	0.032
P242-p240	0.137
P236	***-0.136***
P235	0.088
P234	***0.273***
P233-p230	0.126
p147	-0.019
p133	***-0.351***
p128	0.109
p120	0.116
p096-p093	0.185
p090	0.11612

Bold numbers: major contributing factors defined as greater or smaller than Mean+StandardDeviation, or Mean-StandardDeviation, respectively.

Table S4. Patient clinical and pathological Data for matched recurrent and non-recurrent cases.

		Cases	Status	Epi%	GS	GG1	pT	Pre-Op PSA	Dpsa	Age	DOS	Dof/u
Training Cohort	1	154	BCR	8.01	7	3	2ab	3.1	0.065	62	1/2002	4/2003
		306	non-BCR	15.59	7	3	2ab	5.3	0.126	65	6/2002	6/2011
	2	175	BCR	33.19	7	4	2ab	5.5	0.138	57	1/2002	1/2004
		289	non-BCR	30.00	6	3	2ab	4.4	0.066	60	5/2002	2/2009
	3	252	BCR	32.28	7	3	2ab	6.3	0.183	57	4/2002	9/2005
		302	non-BCR	22.27	7	3	2ab	3.2	0.111	59	6/2002	6/2014
	4	253	BCR	10.17	6	3	2ab	16.3	0.458	67	4/2002	9/2006
		258	non-BCR	7.91	6	3	2ab	3.9	0.095	59	5/2002	10/2014
	5	260	BCR	20.25	7	3	2ab	5.4	0.152	61	5/2002	6/2010
		282	non-BCR	20.80	6	3	2ab	3.3	0.040	64	5/2002	5/2014
	6	265	BCR	18.79	6	3	2ab	3.9	0.066	62	5/2002	2/2003
		329	non-BCR	12.21	6	3	2ab	1.6	0.041	57	7/2002	1/2010
	7	268	BCR	14.67	7	3	2ab	7.1	0.310	51	5/2002	6/2008
		292	non-BCR	13.45	6	3	2ab	0.6	0.010	64	5/2002	4/2014
	8	271	BCR	19.11	6	3	2ab	4.0	0.105	41	5/2002	4/2007
		285	non-BCR	12.70	6		2ab	3.5	0.055	65	5/2002	5/2011
	9	286	BCR	7.97	7	3	2ab	2.7	0.077	58	5/2002	5/2010
		291	non-BCR	11.03	6	3	2ab	4.5	0.032	59	5/2002	11/2013
	10	275	BCR	9.5	7	4	2c	4.9	0.119	50	5/2002	2/2009
		161	non-BCR	10.0	7	3	2c	6.2	0.203	54	1/2002	4/2015
Testing Cohort	1	203	BCR	29.65	6	3	2ab	5.5	0.106	59	2/2002	10/2008
		202	non-BCR	27.82	6	3	2ab	11.0	0.196	55	2/2002	8/2014
	2	209	BCR	1.23	7	4	2ab	5.3	0.183	50	3/2002	1/2003
		247	non-BCR	22.17	7	4	2ab	11.0	0.373	54	4/2002	4/2012
	3	213	BCR	48.75	7	3	2ab	13.0	0.260	51	3/2002	10/2003
		246	non-BCR	39.61	7	3	2ab	1.3	0.051	62	4/2002	5/2014
	4	215	BCR	24.98	6	3	2ab	12.0	0.343	49	3/2002	42008
		615	non-BCR	25.05	6	3	2ab	5.6	0.112	62	7/2003	5/2011
	5	235	BCR	42.23	7	3	2ab	6.3	0.187	57	4/2002	10/2007
		434	non-BCR	52.12	6	3	2ab	8.2	0.200	59	11/2002	6/2014
	6	313	BCR	38.98	7	3	2ab	4.8	0.119	64	6/2002	2/2009
		331	non-BCR	39.62	6	3	2ab	4.8	0.139	61	7/2002	9/2014
	7	314	BCR	28.09	7	3	2ab	4.1	0.110	52	6/2002	5/2005
		346	non-BCR	19.19	6	3	2ab	4.5	0.112	55	7/2002	10/2014

	8	585	BCR	7.80	7	3	2ab	5.4	0.077	63	6/2003	3/2014
		242	non-BCR	15.90	7	3	2ab	8.9	0.150	60	4/2002	12/2009
	9	618	BCR	39.74	7	3	2ab	4.0	0.123	62	7/2003	9/2011
		619	non-BCR	38.19	6	3	2ab	5.8	0.103	69	7/2003	5/2014
	10	333	BCR	47.29	7	3	2c	11.0	0.179	67	7/2002	1/2008
		217	non-BCR	42.98	7	3	2c	1.7	0.065	56	3/2002	5/2010

Table S5. The overall coefficients of Figure 4 in predicting BCR from non-BCR-matched groups for spectral regions are calculated from coefficients for the principal component analysis of all spectral regions and coefficients for canonical analyses by using the four PCs (PC2, PC3, PC8, and PC10) producing the lowest p values (ranging from 0.08 to 0.29) according to the Student t-tests that were conducted on groups of the training cohort. (**Bold** numbers: major contributing factors defined as greater or smaller than Mean+StandardDeviation, or Mean-StandardDeviation, respectively.)

Spectral Regions	Coefficient
p414-410	0.144
p405	0.067
p399-396	-0.017
p394-393	0.120
P378	-0.078
P375	**-0.464**
P374-371	**0.271**
p363	-0.005
p362	0.074
p36	0.017
p334	0.037
p329	-0.042
p327	-0.029
p326	**0.170**
p324	**0.380**
p322	**-0.283**
p320	**-0.400**
p315-310	-0.120
p309-305	-0.165
p303	0.039

p271	-0.034
p268	0.090
p255	0.009
p252	0.138
P245-p243	0.028
P242-p240	-0.007
P236	-0.105
P235	**-0.240**
P234	-0.182
P233-p230	-0.080
p147	-0.159
p133	0.134
p128	-0.042
p120	0.092
p096-p093	-0.032
p090	-0.061
